# A Motion Capture Dataset on Human Sitting to Walking Transitions

**DOI:** 10.1038/s41597-024-03740-z

**Published:** 2024-08-13

**Authors:** Chamalka Kenneth Perera, Zakia Hussain, Min Khant, Alpha Agape Gopalai, Darwin Gouwanda, Siti Anom Ahmad

**Affiliations:** 1 Monash Engineering & Technology Research Hub, School of Engineering, Monash University, Subang Jaya, Selangor Malaysia; 2 Faculty of Engineering, Universiti Putra Malaysia, Serdang, Selangor Malaysia

**Keywords:** Musculoskeletal system, Biomedical engineering, Ageing

## Abstract

Sit-to-walk (STW) is a crucial daily task that impacts mobility, independence, and thus quality of life. Existing repositories have limited STW data with small sample sizes (n = 10). Hence, this study presents a STW dataset obtained via the time-up-and-go test, for 65 healthy adults across three age groups – young (19–35 years), middle (36–55 years) and older (above 56 years). The dataset contains lower body motion capture, ground reaction force, surface electromyography, inertial measurement unit data, and responses for the knee injury and osteoarthritis outcome score survey. For validation, the within subjects intraclass correlation coefficients for the maximum and minimum lower body joint angles were calculated with values greater than 0.74, indicating good test-retest reliability. The joint angle trajectories and maximum voluntary contractions are comparable with existing literature, matching in overall trends and range. Accordingly, this dataset allows STW biomechanics, executions, and characteristics to be studied across age groups. Biomechanical trajectories of healthy adults serve as a benchmark in assessing neuromusculoskeletal impairments and when designing assistive technology for treatment or rehabilitation.

## Background & Summary

Sit-to-walk (STW) is a fundamental weight-bearing transition that plays a pivotal role in ensuring mobility and independence during activities of daily living (ADLs). It is defined as a fluid merging of sit-to-stand and gait^1^, yet literature has conventionally leaned towards sit-to-stand transitions. However, usually after standing the end goal is walking, making STW a common and functionally significant ADL with sit-to-stand being a subset of STW. This view is shared by recent literature, such as Perera *et al*.^2^, van der Kruk *et al*.^3^ and Rousanoglou *et al*.^4^. As a result, there is an interest to study human motion capture (Mocap) data to analyse STW biomechanics (such as joint torques, loads, and muscle forces), characteristics, and execution strategies^2,5^. Investigating healthy adult STW biomechanical trajectories facilitates a benchmark that can be used to inform and assess neuromusculoskeletal impairment, track recovery progress in rehabilitation, determine the efficacy of a treatment compared to subjective clinical tests^6,7^, and in the design of assistive technology for impaired motion.

Literature does indeed contain open access Mocap repositories covering multiple motions, as presented in Table 1. For instance, the Berkley Multimodal Human Action Database^8^ presented 11 highly dynamic upper and lower body motions, inclusive of sitting down and standing up, but not STW transitions. It had a small sample size and narrow age range of 11 young adults and one older adult. Similarly, the continuing KIT Whole-Body Human Motion Database^9^ contained 2925 motion experiments and 234 subjects with object interaction tasks, but not STW. Furthermore, the database presented by Camargo *et al*.^10^ focused on four locomotion based activities, excluding STW, and only considered 22 healthy young adult subjects. Conversely, the recent Asian-centric human movement database^11^ had 12 daily tasks (such as gait, key turning, or towel folding) and was inclusive of STW transitions obtained via the timed-up-and-go (TUG) test. In this landmark database, a wide age range of volunteers between 21–80 years were considered, however only a small sample size of 10 subjects were made publicly available, which poorly represents a population. Yet, this database has over 5600 accesses and 10 citations (Scientific Data, Springer Nature), showing a growing need for STW Mocap data.

**Table 1:** Overview and comparison of selected motion capture datasets.

Database	Motions	Sensors or Systems	Age Range	Sample Size
Berkly multimodal human action database^8^	Highly dynamic 11 upper and lower body activities.	Mocap, Kinect, accelerometers, and an audio system.	20 to 30 years	11 healthy adults
KIT whole-body human motion database^9^	2925 object-oriented experiments.	Mocap, video and object information inclusive of 3D models and images.	—	234 adults
Lower limb dataset by Camargo *et al*.^10^	Treadmill and level-ground walking with ramp and stair ascent/decent.	Mocap, force plates, IMUs, and SEMG.	19 to 24 years	11 healthy adults
Asian centric human movement database^11^	12 upper and lower body ADLs inclusive of STW.	Mocap, force plates and load cells, with object detection modules, and a dynamometer.	21 to 80 years	10 healthy adults
A Mocap dataset on human sitting to walking transitions (our)^31^	STW transitions with quiet sitting and gait for the lower body.	Mocap, force plates, IMUs, SEMG, and KOOS survey.	19 to 73 years	65 heathy adults

Thus, this study seeks to complement existing STW databases by capturing a large sample of 65 healthy subjects with a wide age range from 19–73 years, spanning young, middle-aged, and older adult age groups. From this, the age-wise variation in STW biomechanics, characteristics, and execution strategies can be captured, while being statistically generalizable to a population^12–14^. The study herein presents lower body STW Mocap, ground reaction force (GRF), surface electromyography (SEMG), and inertial measurement unit (IMU) data, in addition to responses for the knee injury and osteoarthritis outcome score (KOOS) survey. STW transitions were obtained from quiet sitting till gait via the forward movement of the TUG test, which is a standard clinical assessment^15^. However, the TUG test presented in this paper does not account for mediolateral or rotational trunk movement to stand up and walk in an alternate direction and is therefore a limitation of this test.

## Methods

### Participants

Participants were community dwellers who were recruited through word of mouth and electronic advertisements. This study consists of 65 healthy subjects, categorized by three standard age groups based on chronological ageing. These were (1) young adults from 19–35 years, (2) middle-aged adults from 36–55 years, and (3) older adults from 56 years onwards^16,17^. The average age, weight, height, and male/female distribution with respect to age group are presented in Table 2. All participants were healthy adults that could stand and walk comfortably without external aid and showed no movement impairment. Consequently, the exclusion criteria were: (1) declared physical, musculoskeletal, or neurological impairments that affect movement or daily tasks, (2) declared mental health issues that affect daily tasks, and (3) current pregnancy. A list of individual subject demographics is provided along with the dataset, as discussed in the Data Records section below. All data collection experiments were conducted in the Motion Capture Laboratory at Monash University Malaysia and the methodology of this study was reviewed and ethically approved by the Monash University Human Research Ethics Committee, under the project number 32328. All subjects provided written informed consent.

**Table 2:** Subject descriptives based on age groups.

Subject Descriptives			
Young Adults (19–35 Years)			
	Range	Mean ± Standard Deviation	
Age (Years)	19–33	24.19 ± 4.34	
Weight (kg)	38.60–107.90	64.12 ± 17.04	
Height (cm)	144.50–182.00	165.33 ± 9.45	
**Middle-aged Adults (36**–**55 Years)**			
Age (Years)	36–50	42.13 ± 4.55	
Weight (kg)	48.65–95.25	67.94 ± 15.85	
Height (cm)	144.80–179.40	164.46 ± 10.47	
**Older Adults (56**–**73 Years)**			
Age (Years)	57–73	64.00 ± 4.43	
Weight (kg)	45.20–84.10	59.38 ± 10.70	
Height (cm)	146.80–166.80	156.94 ± 6.07	
**Subject Distribution by Biological Sex**			
	**Young Adults (19**–**35 Years)**	**Middle-aged Adults (36**–**55 Years)**	**Older Adults (56**–**73 Years)**
Male	19	9	4
Female	13	6	14
Total	32	15	18

### Instrumentation

**Fig. 1:**
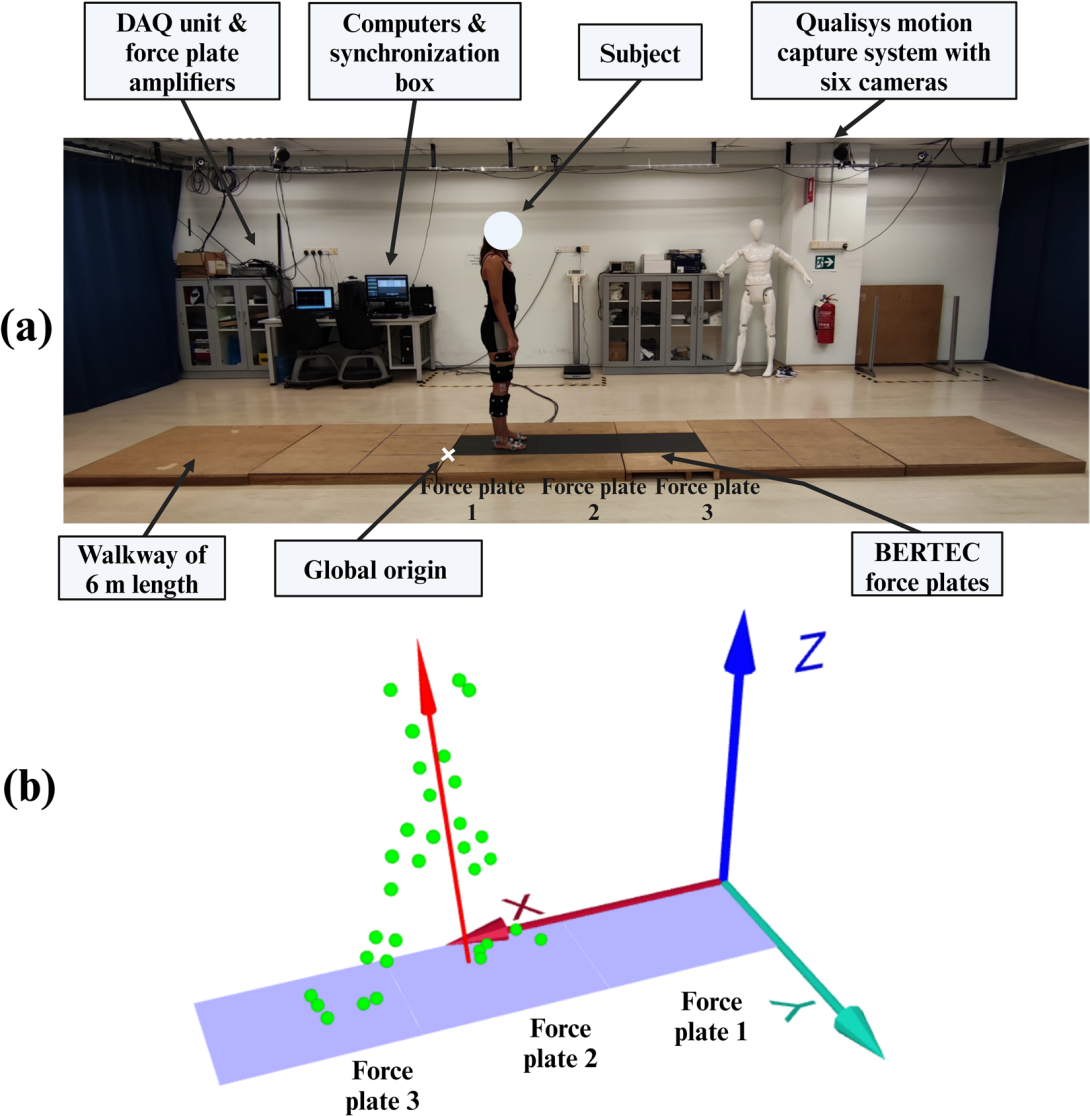
(**a**) The motion capture laboratory setup with a 6 m wooden walkway, six Qualisys cameras, three BERTEC force plates and amplifiers, a data acquisition (DAQ) unit, a Delsys Trigno surface electromyography (SEMG) and inertial measurement unit (IMU) system, Delsys Trigger Module, and computers. The global laboratory origin is marked by a white X at the edge of force plate 1. (**b**) Shows the three force plates aligned serially and the global laboratory coordinate axes as visualized from Qualisys Track Manager (QTM).

The laboratory setup for data collection (Fig. 1) consisted of a Qualisys Mocap system, BERTEC force plates, Delsys Trigno SEMG and IMU sensors, a flat 6 m wooden walkway to perform STW, and the required computer systems for each instrument. A pushbutton on the Delsys Trigger Module was used to start each data collection trial and would simultaneously activate the Delsy Trigno and Qualisys Mocap systems, which would then trigger the force plates, thus time synchronising all instruments. Moreover, the global laboratory coordinate origin is at the edge of force plate 1 (Fig. 1), with the X-axis in the anteroposterior direction, the Y-axis in the mediolateral direction, and the positive Z-axis pointing vertically upwards.

#### Optical motion capture

**Fig. 2:**
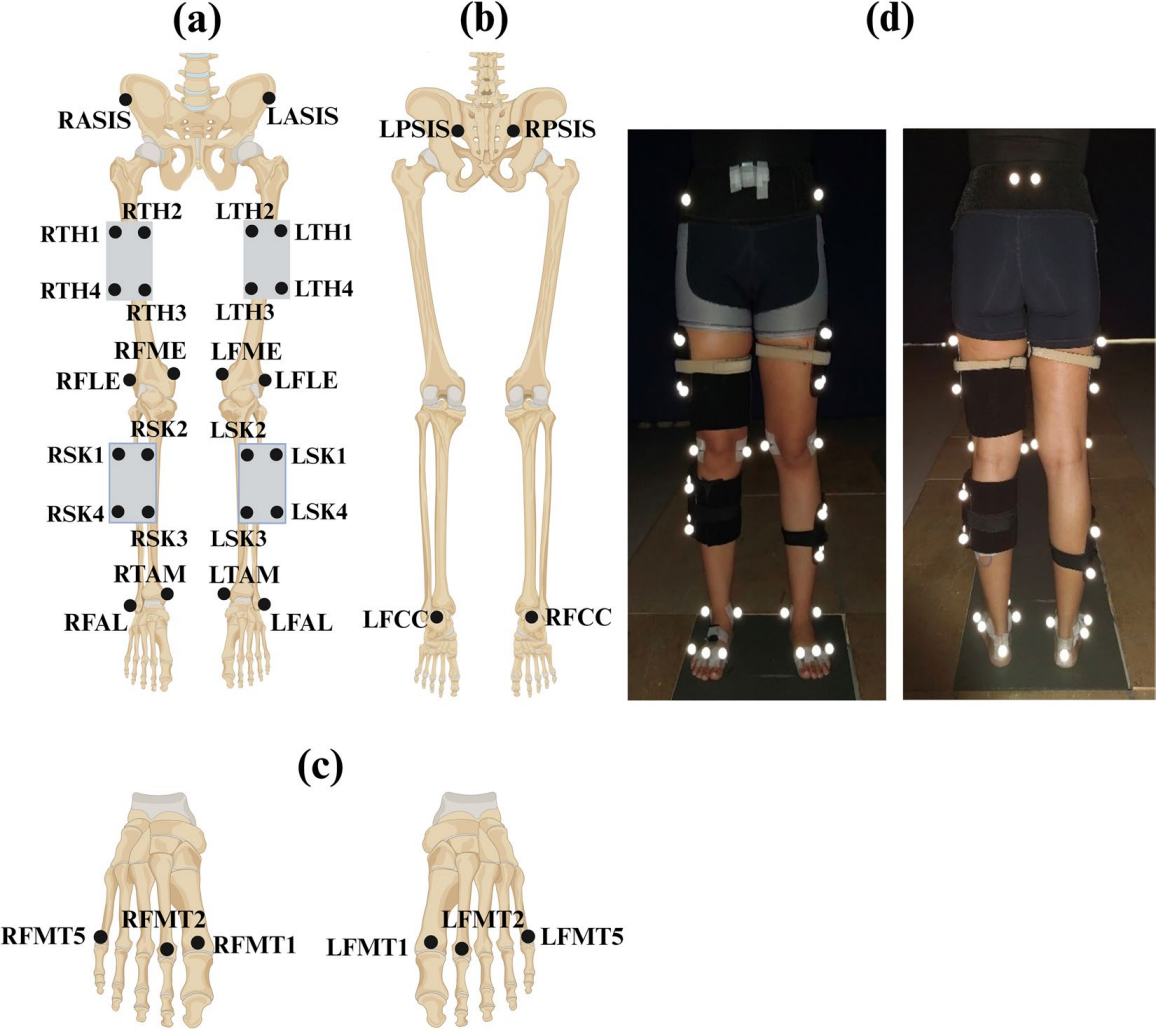
Lower body reflective motion capture marker placement where (**a**) is the anterior view, (**b**) is the posterior view, and (**c**) is the anterior view of the feet. (**d**) Shows the anterior and posterior views of marker placement on a test subject. The black bands on the foot were placed over the Delsys Trigno surface electromyography (SEMG) and inertial measurement unit (IMU) sensors.

The optical Mocap system consisted of six ceiling mounted Oqus Qualisys cameras (Qualisys AB, Sweden), to collect 3D Mocap marker (kinematic) data. The cameras had a 56° horizontal field of view while Qualisys Track Manager (QTM) served as the software interface for camera configuration, recording Mocap trials, and for synchronization with the force plates and Delsys sensors. Mocap data was collected at a sampling frequency of 200 Hz and the calibration volume of the cameras was centred along the wooden walkway, encompassing the three force plates. Calibrations were verified by ensuring wand standard deviation was less than 1 mm, without gaps in the visualized volume^18^. Moreover, 36 passive retro-reflective Mocap markers (12.5 mm diameter) were placed along the subject’s lower-body for motion tracking. Markers were placed following the Calibrated Anatomical System Technique (CAST)^19^ and with reference to Visual 3D^20^, as illustrated in Fig. 2.

#### Force plates

Three BERTEC (Ohio, United States) FP4060-07 force plates were placed serially along the laboratory, centred within the wooden walkway (Fig. 1), to capture GRF and center of pressure (COP) during STW. The force plates had dimensions 600x400x75 mm and a sampling frequency of 1000 Hz. The origin for the COP of the three force plates correspond to the global laboratory origin (Fig. 1) and the plates were placed flush against the walkway for a flat walking surface.

#### Surface electromyography and inertial measurement units

**Fig. 3:**
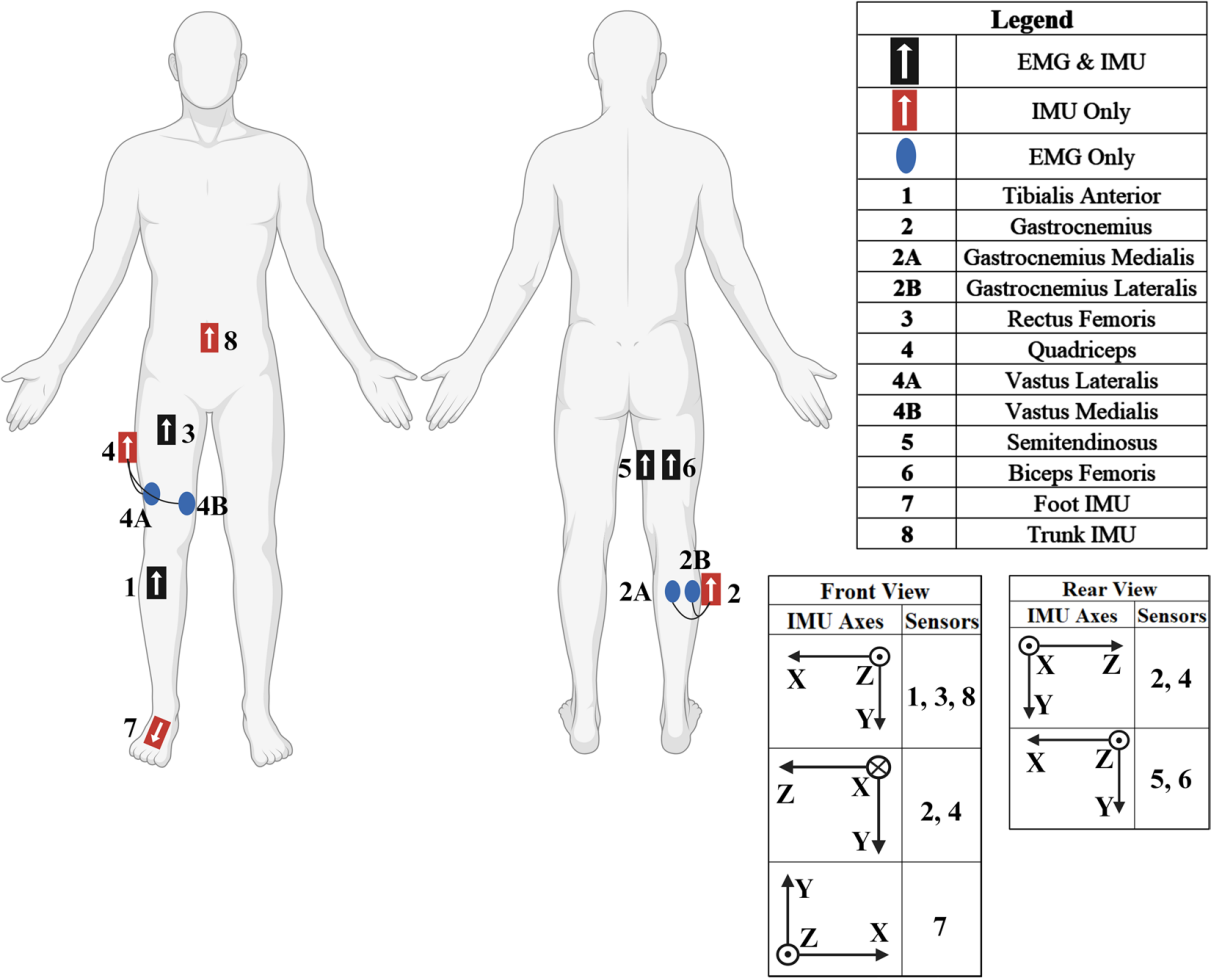
Sensor placement of the Delsys Trigno Avanti (black) and Trigno Duo (red) for surface electromyography (EMG) and inertial measurement unit (IMU) readings. The targeted muscles and IMU orientation axes are provided in the legend.

A wireless Delsys Trigno system (Delsys Inc., Boston, USA) was used to record the SEMG and IMU data, consisting of six Trigno Avanti and two Trigno Duo sensors. Each sensor collects both SEMG and IMU data simultaneously, with the Trigno Duo recording SEMG from two muscles. However, sensors seven and eight (in Fig. 3) were designated for only collecting the IMU data of the foot and trunk. Each IMU reading has six channels with three for the axes of acceleration (accelerometer) and three for the axes of angular velocity (gyroscope). The sampling frequencies for SEMG were 1259 Hz and 1778 Hz for Avanti and Duo, respectively. Similarly, all IMU channels were sampled at 148 Hz for the Avanti, while for the Duo, the accelerometer (ACC) was sampled at 963 Hz and the gyroscope (GYRO) at 741 Hz. These maximum sampling frequencies were fixed by the Delsys Trigno System, and a recommendation on handling the varying sampling frequencies is presented in the Data Preprocessing section below. Due to these varying frequencies, the provided CSV files will be of different lengths for the SEMG and IMU time series.

SEMG was used to record the muscle activity from eight major muscles on the dominant leg, per subject. The targeted muscles were the *Tibialis Anterior*, *Gastrocnemius Lateralis* and *Medialis*, *Rectus Femoris*, *Vastus Lateralis* and *Medialis*, *Biceps Femoris* and *Semitendinosus*. These muscles include the major lower extremity muscle groups and were selected based on their involvement in hip and knee extension, which are the primary drivers of STW^21^. Furthermore, these sensors were attached to the subject through palpation, following the guidelines and muscle placement locations as recommended by the SENIAM (Surface Electromyography for the Non-Invasive Assessment of Muscles) project^22^, and is illustrated in Fig. 3^22^.

#### KOOS survey

The KOOS survey is a widely used tool to evaluate the subjective perception of knee function. It is a standardised questionnaire consisting of 42 items that asses five outcomes: pain, symptoms, ADLs, sport and recreation activities, and knee-related quality of life. Each question is answered on a five-point Likert scale, with zero indicating no difficulty and four indicating extreme difficulty in performing a task. Subject responses are used to calculate the scores for each outcome separately, by adding the individual scores of the questions included in each domain. The scores are then transformed to a percentage, with zero indicating extreme knee difficulties and 100 indicating no knee difficulties. Therefore, KOOS provides a quantitative measure of a patient’s knee function and the impact of knee problems in their life. The KOOS scores can be used to identify associations with other biomechanical parameters derived from Mocap, providing insights into impaired knee function^23^. Further information on calculating the KOOS score can also be found in reference^23^ and several online KOOS calculators such as OrthoToolKit are available.

### Data collection procedure

#### Prior to data collection

Volunteers were briefed and provided an explanatory statement of the study that covered the purpose and procedures involved. It was explained that the recommended attire for the experiment was short, dark, tight-fitting clothing (such as sportswear) without any reflective accessories such as watches or jewellery. Upon acceptance of the experimental protocol an appointment with the preferred date was set.

On the day of the experiment, on arrival, written informed consent was obtained along with subject details such as age, height, weight, and dominant leg (defined as kicking leg). Participants were screened on their dress code to ensure that there was no movement of clothing and interference with the sensors or markers during data collection. The KOOS survey was then conducted and saved in a de-identified manner, based on a running subject number. The Mocap markers were placed on the subject’s lower body and secured using a combination of Velcro bands and surgical tape (see Fig. 2). The marker locations correspond to the anatomical lower body joints as listed in Supplementary file 1. Only the hip anterior and posterior markers (ASIS and PSIS) were placed over clothing (to protect modesty) and secured firmly to the body using a Velcro band.

**Fig. 4:**
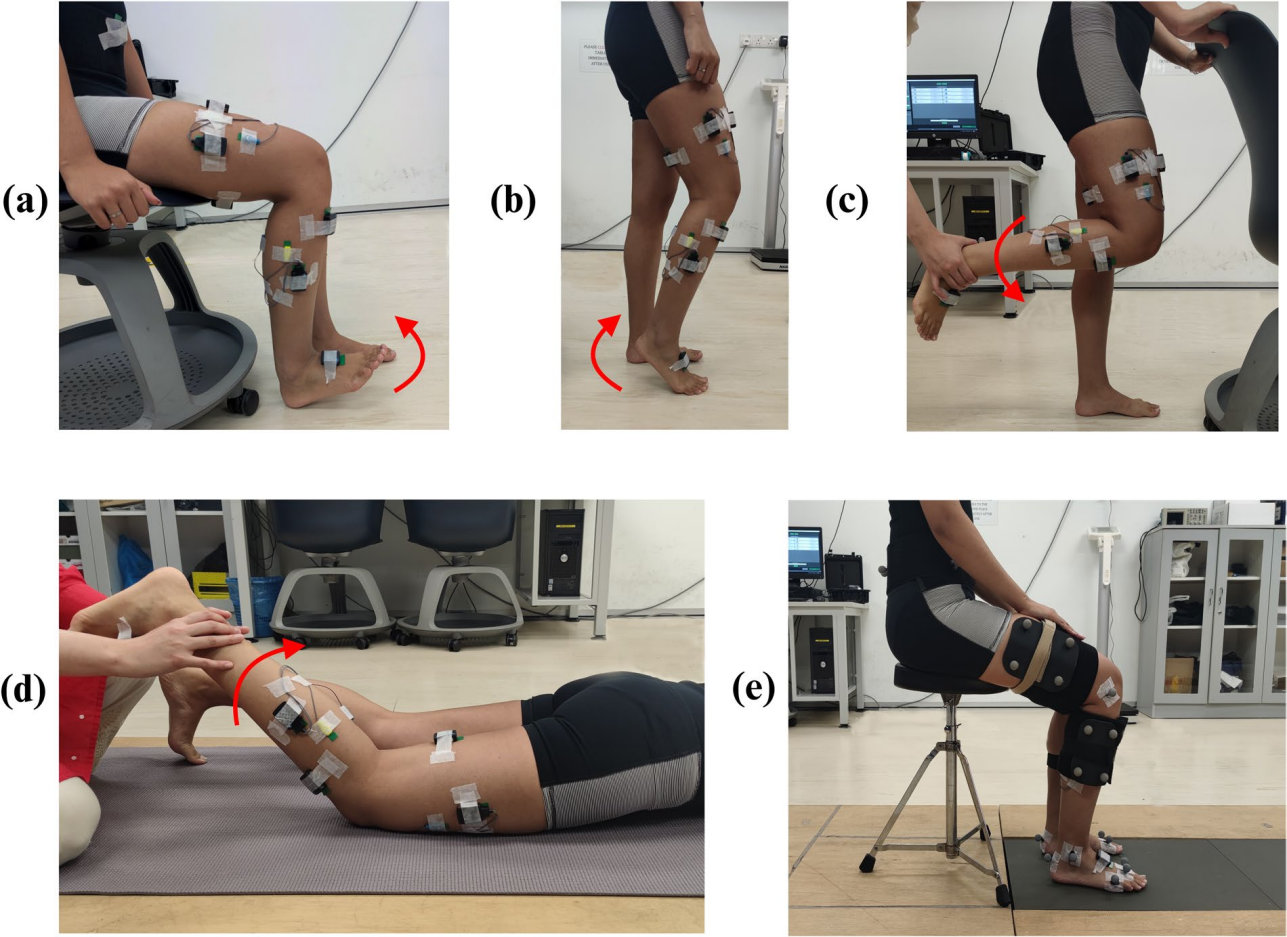
Conducting the maximum voluntary contraction (MVC) for the (**a**) Tibialis Anterior, (**b**) Gastrocnemius, (**c**) Quadriceps and (**d**) Hamstrings, as recommended by the SENIAM project. Additionally, (**e**) shows the initialised sitting position for the sit-to-walk dynamic trials.

Following this, the targeted muscles on the dominant leg (Fig. 3) were located through palpation, as recommended by the SENIAM project^22^. The surface of the skin around the muscles were cleaned using an alcohol swab and the Delsys Trigno SEMG and IMU sensors were placed using double-sided Trigno sensor adhesives with surgical tape. Black Velcro bands were also placed around the sensors and subject’s leg (Fig. 2) to ensure good sensor contact with the skin and prevent slipping during data collection. Thereafter, a maximum voluntary contraction (MVC) was performed for each muscle group, with subjects maximally contracting their muscles, as detailed by the SENIAM project^22^. MVCs were performed for the *Tibialis Anterior*, *Gastrocnemius*, Quadriceps, and Hamstrings, as illustrated in Fig. 4.

#### During data collection

A static Mocap trial was first recorded per subject with participants being asked to stand in a T-pose on force plate one. This is used when scaling a musculoskeletal model^24^. Subsequently, STW dynamic trials were performed via the TUG test^15^ with Mocap, SEMG, and IMU being measured. The medial knee and ankle Mocap markers (FME, TAM) were also removed to avoid knocking during STW. Subjects were initialized in a seated position on an armless, backless, height adjustable stool, with their hands on their lap, both feet on force plate one, and knees at approximately 90° (see Fig. 4). On cue from the experimenter, the subject then stood up and walked 3 m, turned around, walked back, and sat down, as quickly as comfortably possible. The starting posture was controlled for all subjects throughout the experiment, however once STW began subjects employed their natural movement pattern without any constraint to arm or leg motion. Five repetitions were obtained per subject and STW was extracted from the forward portion of this test.

#### After data collection

After the experiment concluded, all items and sensors were removed from the subject and a token of appreciation was provided. The recorded Mocap, SEMG, and IMU data along with respective subject details were stored in de-identified manner based on the subject number.

### Data preprocessing

The Mocap files in this study were exported from QTM into C3D format and contain both marker trajectories and force plate readings (GRFs, COP, and moment). Similarly, the SEMG and IMU files per trial were collectively exported from the Delsys Trigno system into a single CSV file. All data files (Mocap, SEMG and IMU) were cropped to the same STW time periods, from quiet sitting till the subject left force plate three during gait. Quiet sitting occurs before STW movement initiation, which is denoted by the first change in vertical GRF or the start of an anterior increase in horizontal center of mass velocity^1,2^. As such, STW was obtained from the forward portion of each TUG trial in the dataset. Subsequently, the Mocap marker trajectories were labelled following the CAST convention (see Supplementary file 1), while any missing trajectories were gap filled (interpolation). External reflections or disturbances (such as obstructing clothes or hand movements) could cause the Mocap cameras to misinterpret marker locations and trajectories due to noise. In these instances, incorrect marker trajectories were discarded.

The data presented in this study was not filtered to preserve the raw signals obtained from the experiments. Yet, it is recommended to filter the data prior to analysis - for minimizing noise, motion artifacts and smoothing, which reduces fluctuations in the data and produces an average representation to better identify trends or patterns. As such, for Mocap it is recommended to use a zero-lag second-order Butterworth lowpass filter at cut-off frequencies less than 10 Hz^25,26^. Likewise, for SEMG a zero-lag second-order Butterworth bandpass filter with cut-off frequencies from 20 to 400 Hz can be used^27^. Filter cut-off frequencies should be tailored to the relevant application and can be chosen by performing a Fast Fourier Transform to analyse the occupied signal bandwidth.

Moreover, due to the varying sampling frequencies between Mocap (200 Hz), SEMG (1259 Hz for the Avanti and 1778 Hz for the Duo) and IMU (148 Hz for the Avanti, 963 Hz for ACC and 741 Hz for GYRO in the Duo), it is recommended to downsample or time normalise the signals (currently not performed in this dataset). These techniques were utilised in references^21,28^ and allows the data to be interpreted or analysed on a consistent time scale while reducing data redundancy. Also, for SEMG an envelope (measured in mV) can be obtained through full wave rectification and low pass filtering with an approximate cut-off frequency of 6 Hz^26^. These envelopes can then be normalised and represented as a percentage of the maximum muscle activity, through division by the peak MVC per subject^29^. Normalisation reduces the effects of electrode displacement during motion, muscle crosstalk, sweat, temperature, and subcutaneous fat. This process minimizes inter-subject variability allowing for a consistent comparison of SEMG signals across muscles, trials, and subjects^30^.

## Data Records

The dataset presented in this study is publicly available in the Bridges Repository by Monash University^31^, with the identifier: 10.26180/24515092.v4. The Mocap marker and force plate data for STW are stored in C3D format, with additional information such as sampling rates and system settings provided under the header information and parameter groups. Alongside this, the SEMG and IMU data are presented in CSV format, with SEMG, ACC (in X, Y, and Z axes), and GYRO (in X, Y, and Z planes) data columns for each sensor. The header information also includes the corresponding muscle group, sampling frequency, and sensor number. Within each subject folder the KOOS survey response is presented as a PDF, while subject details containing sex, age, weight, height, and dominant leg are given in a single global CSV file. Furthermore, the dataset is approximately 1.03 GB in size and a ‘README.txt’ file is provided.

With respect to the dataset naming convention, each subject’s data is stored in a unique folder numbered from S01 to S65. Each folder contains two subfolders, the first for Mocap data and the second for SEMG and IMU. Under the ‘Mocap’ folder, the static trial is provided along with the five STW repetitions labelled from ‘stw1’ to ‘stw5’. Likewise, under the ‘EMG and IMU’ folder, data for the STW repetitions are also labelled from ‘stw1’ to ‘stw5’. Further, MVC files corresponding to the three muscle groups are provided with names ‘mvc_hamstrings’, ‘mvc_quadriceps’ and ‘mvc_shank’. The hamstrings include the *Biceps Femoris* and *Semitendinosus*; the quadriceps comprise of the *Rectus Femoris* and *Vastus Medialis/Lateralis* and the shank contains the *Tibialis Anterior* and *Gastrocnemius Medialis/Lateralis*.

## Technical Validation

**Fig. 5:**
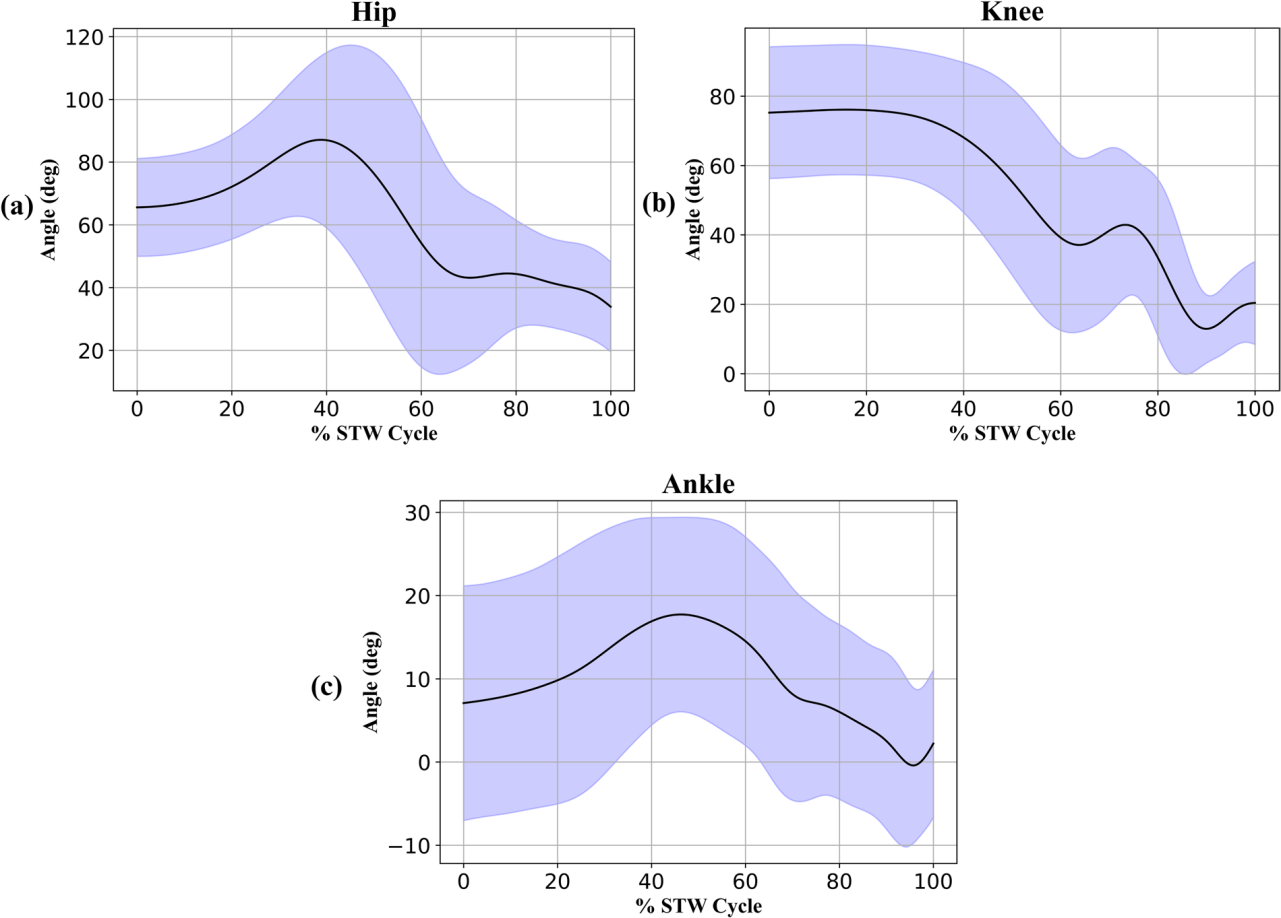
(**a**) Hip, (**b**) knee, and (**c**) ankle joint angle trajectories as a percentage of the sit-to-walk cycle. The solid black line shows the average joint angles (n = 65), while the shaded blue region represents two standard deviations, corresponding to approximately 95% of the subject group joint angles.

For validation of the collected Mocap data, the hip, knee, and ankle joint angles were derived, and the within subjects intraclass correlation coefficient (ICC) for the maximum and minimum joint angles (Table 3) were calculated. For each subject, the raw Mocap marker data was filtered using a second-order Butterworth lowpass filter at a 5 Hz cut-off frequency, as described in the Data preprocessing section. OpenSim 4.2^32,33^ was used to derive the lower-limb joint angles using the publicly available Gait2392 Musculoskeletal model^34^. Here, static trials were used to perform scaling which matches the subject anthropometry to the model by minimising the distance between the experimental and virtual (model) markers. Scaling produces a subject specific musculoskeletal model and was validated by ensuring the maximum and root mean square marker error was kept below 2 cm and 1 cm respectively, as recommended by OpenSim^24^. Following this, inverse kinematics was performed to obtain the hip, knee, and ankle joint angles, normalised to 0° when standing upright (Fig. 5). The maximum and minimum joint angles for the five repetitions of each subject were considered when calculating the ICC. For this, SPSS Statistics (IBM) was used with a single-measurement, absolute-agreement, two-way mixed-effects model, and a 95% confidence interval^35^.

**Table 3:** Intraclass correlation coefficients (ICC) for the maximum and minimum hip, knee, and ankle joint angles.

Joint Angle ICC	Hip		Knee		Ankle	
	Max	Min	Max	Min	Max	Min
Single Measures	0.90	0.81	0.84	0.74	0.79	0.80
95% Confidence Interval	0.86–0.93	0.74–0.87	0.78–0.89	0.66–0.82	0.71–0.85	0.73–0.86

Table 3 presents the ICC results for the three lower body joints, with all values being greater than or equal to 0.74. The lower bound confidence intervals are greater than 0.71 except for the minimum knee which displays moderate strength. Overall, the ICCs reflect a good degree of correlation within subject measurements and showcase good test-retest and intra-rater reliability^35^. Additionally, the joint angle trajectories (Fig. 5) are comparable with existing literature for STW transitions^3,21^ and produce a close match in the overall trends and ranges. These results validate the Mocap data collected in the experiment.

Considering SEMG, electrodes were positioned following the guidelines established by SENIAM^22^, where sensor placement was verified by visually inspecting the signal quality during muscle activation movements and then adjusted as required. To validate the SEMG data collected in this study a comparative analysis between muscle MVCs was performed, based on a publicly accessible dataset from literature by Hu *et al*.^36^. MVCs were selected as no current database contains SEMG data for STW transitions. The dataset by Hu *et al*.^36^ consists of MVCs from 10 young adults (age: 25.5 ± 2 years, height: 174 ± 12 cm, and weight: 70 ± 14 kg), hence, only the MVCs from young adults in this study were used for the comparison. For both datasets the MVCs were bandpass filtered between 20 and 450 Hz, rectified to remove negative values, and lowpass filtered with a cutoff frequency of 6 Hz to obtain linear envelopes. Pearson’s correlation coefficient was then computed between the averaged MVC envelopes of the two datasets^37^, for six muscles as presented in Table 4.

**Table 4:** Correlation of muscle maximum voluntary contractions (MVCs) between existing literature^36^ and the current dataset.

Muscles Compared	Pearson’s Correlation of MVCs
*Tibialis Anterior*	0.91
*Gastrocnemius Medialis*	0.47
*Rectus Femoris*	0.63
*Vastus Lateralis*	0.97
*Biceps Femoris*	0.89
*Semitendinosus*	0.88

The results (Table 4) show a strong correlation (greater than or equal to 0.88) between the muscle MVCs of both datasets, except for the *Rectus Femoris* and *Gastrocnemius Medialis* which have moderate strength. Overall, this demonstrates a close match in trend between existing literature^36^ and the current dataset, thus validating the quality of the SEMG data collected in this experiment. The lower correlations of the *Rectus Femoris* and *Gastrocnemius Medialis* can be attributed to several factors which include the anatomical and functional variability of the muscles, changes in electrode placement, movement specificity, and population or demographic differences between the two studies^37^.

## Usage Notes

This dataset comprises Mocap, GRF, IMU, and SEMG data that can comprehensively analyse lower limb motion during STW. Compared to existing STW data^11^, this dataset offers a larger sample size (n = 65) over a wide age range (19–73 years), with multiple measured biomechanical quantities. This data would aid in understanding STW execution strategies and biomechanics like joint dynamics and muscle activation in healthy adults^2,38^. In neuromusculoskeletal modelling, SEMG data plays a crucial role in characterising subject-specific muscle activation patterns. By incorporating this data, it is possible to accurately estimate internal biomechanics like muscle forces and subsequently joint loads, which can provide valuable insights into understanding the incidence and progression of joint degeneration^39^. Such findings, using healthy adult Mocap, IMU, and SEMG data, in addition to knee health information via KOOS responses, can be used in rehabilitation to set a benchmark. This enables to assess and identify neuromusculoskeletal impairments, monitor recovery progress, and develop treatment plans for joint injury or degradation due to factors such as osteoarthritis^7,23^.

Moreover, biomechanical data can be used in the design-evaluation process of assistive devices and their core technologies. For instance, healthy adult joint torque trajectories can serve as a reference or baseline when providing assistive torque for impaired motion. The 65 subject sample allows for training neural networks to estimate assistance or movement intention and trajectories, which are applicable for the control architectures of assistive devices^40,41^.

### Supplementary information

Description of reflective motion capture markers attached to the subject's lower body.

## Data Availability

No custom codes were created or utilized in this study.
